# Application of the transposon-associated TnpB system of CRISPR-Cas in bacteria: *Deinococcus*

**DOI:** 10.3389/fmicb.2025.1604032

**Published:** 2025-09-15

**Authors:** Zi-Qi Yang, Mei-Ju Li, Faizan Ahmad, Chun-Zhi Jin, Taihua Li, Feng-Jie Jin, Kee-Sun Shin, Long Jin

**Affiliations:** ^1^Co-Innovation Center for Sustainable Forestry in Southern China, College of Ecology and Environment, Nanjing Forestry University, Nanjing, China; ^2^College of Forestry and Grassland, Nanjing Forestry University, Nanjing, China; ^3^Microbiome Convergence Research Center, Korea Research Institute of Bioscience and Biotechnology (KRIBB), Daejeon, Republic of Korea

**Keywords:** *Deinococcus*, *Deinococcus radiodurans*, CRISPR/Cas, TnpB, transposon

## Abstract

*Deinococcus radiodurans* is one of the most radioresistant organisms found on Earth to date, showing extreme resistance to damage factors such as UV, drought, and mutagens, and is of great interest to scientists around the world. It was determined that the TnpB protein from *D. radiodurans* ISDra2 functions as an RNA-guided endonuclease, serving as a functional ancestor for the widely used CRISPR-Cas endonucleases. The CRISPR-Cas system is an “acquired immune system” found in most Bacteria and Archaea, and used in a wide range of biological and medical research fields. Cas12f is the smallest RNA-directed nuclease that is currently known and possesses unique characteristics. There has been extensive research conducted on the origin, classification, and mechanism of action of CRISPR-Cas12f, as well as its application in the field of gene editing. TnpB, as the protein closest to Cas12f in the evolutionary tree, has the potential to be used as a new micro-editing tool. Systematic studies have been conducted on it to develop smaller volumes of precision gene editing and treatment tools. In this review, the research progress, mechanism, and application of TnpB protein in *D. radiodurans* were reviewed. In addition, the classification of CRISPR-Cas system and the application and function of CRISPR-Cas12f in gene editing are also introduced and summarized.

## 1 Introduction

*Deinococcus radiodurans* was first isolated from radiation-sterilized canned meat in 1956 by the American scientist Anderson et al. (Anderson et al., 1956; Jung et al., 2017). It is one of the most radiation-resistant organisms found on Earth to date, and therefore is known as the world’s most resistant organism (Anderson et al., 1956). The most remarkable feature of *D. radiodurans* is that it can tolerate a radiation dose of 15 kGy during its stable growth period, which is more than 250 times the radiation resistance of *E. coli* and 3,000 times that of humans (Cox and Battista, 2005). Because of its extreme resistance characteristics, *D. radiodurans* is of great importance in the study of radiation resistance mechanism, environmental remediation and tumorigenesis, and is therefore of great interest to scientists worldwide as a promising microbial resource for exploitation (Desai et al., 2011). *Deinococcus* is capable of withstanding extreme radiation and stress by means of its survival mechanisms, which are supported by DNA repair systems, genomic features, and unique cell wall structures. The condensed genomes of *Deinococcus* contain repetitive sequences that are involved in the degradation of damaged DNA. The cell envelope of certain *Deinococcus* strains is composed of six layers, each of which is composed of distinct components such as lipids, proteins, and carbohydrates (Jin et al., 2019; Liu et al., 2023). *D. radiodurans* has a very special cell wall structure. According to the structure and composition of its cell wall should belong to Gram-negative bacteria, but because of the thick peptidoglycan layer in the cell wall, it is difficult to decolorize after crystal violet staining, and the Gram stain is positive. Meanwhile, the cell wall has an outer membrane but lacks the teichoic acid contained in ordinary Gram-positive bacteria. This multi-layer cell wall structure has a certain blocking effect on ionizing rays and ultraviolet rays, which can slow down the damage caused by radiation to the bacteria, and play a role in supporting and protecting the cells (Sharma et al., 2024).

*Deinococcus radiodurans* not only exhibits extraordinary radiation resistance but also possesses the remarkable ability to fully restore its genome from hundreds of double-strand breaks (DSBs) and thousands of single-strand breaks (SSBs). Therefore, studying the DNA damage repair mechanisms in this bacterium is of significant importance–not only for elucidating the molecular basis of DNA repair but also for applications in environmental protection, bioremediation, human health, and even the exploration and utilization of extraterrestrial environments. This has made it a major research focus in recent years (Mishra et al., 2021; Sharma et al., 2025). The chromosome structure is also special. During the stationary growth phase, the nucleoid of *D. radiodurans* presents a very compact circular structure. Researchers believe that this dense ring structure can prevent the fragments formed after double-strand breaks from dispersing during the repair process, thus facilitating the repair of DNA damage and providing passive defense against radiation (Zhang et al., 2009; Mishra et al., 2021). However, this hypothesis has also been disputed based on studies with *E. coli* (Zimmerman and Battista, 2005). Nevertheless, a highly dense genomic structure that prevents the diffusion of damaged DNA and thus facilitates the search for repair templates (Kota and Misra, 2006; Rajpurohit et al., 2013; Kota et al., 2014) is still a reasonable explanation. *D. radiodurans* has an efficient DNA damage repair mechanism. The most obvious feature of *D. radiodurans* is that it can withstand gamma rays of more than 15 kGy and repair up to thousands of DNA double-strand breaks (Redox Signals) produced by radiation within a dozen hours, reconstructing the genome without mutation and without affecting its viability (Minton and Daly, 1995; Bihani et al., 2018). The known DNA repair methods of *D. radiodurans* include five types: base excision repair, direct damage repair, nucleotide excision repair, base mismatch repair and recombination repair (Timmins and Moe, 2016; Sharma et al., 2025), among which DNA recombination repair includes homologous recombination (HR), non-homologous end joining (NHEJ), single-strand end annealing (SSA), and synthesis-dependent single-stranded end joining (SDSA). SSA and SDSA, as well as a new DNA recombination repair pathway, extended synthesis-dependent strand annealing (ESDSA), were proposed by Zahradka et al. (2006). Before DNA damage, the DNA template has some DNA duplexes as patches, and after DNA damage, the newly synthesized DNA duplexes link the patched duplexes to complete the repair (Bentchikou et al., 2010). Its diverse DNA damage repair mechanism is expected to be further exploited and developed as an effective tool for gene editing. The CRISPR-Cas system is a defense mechanism widely present in bacteria and archaea. Currently, genome editing tools developed based on this system have achieved targeted editing in numerous species. However, their protein sizes all exceed 1,000 amino acids, which hinders delivery efficiency. The TnpB protein from transposon families in *D. radiodurans* has been identified as the ancestor of Cas12. These proteins are collectively referred to as Obligate Mobile Element-Guided Activity (OMEGA) proteins, with their guide RNA called ωRNA. Consequently, the OMEGA system has emerged as a research hotspot in the field of gene editing (Sasnauskas et al., 2023). Further exploration of *D. radiodurans*’ efficient DNA repair mechanisms and natural transformation capabilities may lead to the development of precision gene editing tools suitable for extreme environments, thereby promoting innovative applications of this bacterial strain in broader biotechnological scenarios.

In the past, radioresistance of *D. radiodurans* was mainly attributed to its resistance and repair mechanisms against DNA damage, and a lot of research has been conducted in this area. However, as research progressed, it was discovered that *D. radiodurans* also has a superb oxidative defense system, which is another important reason for its extreme radioresistance (Kota et al., 2014; Maurya et al., 2018; Misra and Rajpurohit, 2024). It was found that along with the direct damage to DNA and proteins caused by ionizing radiation, a large number of highly cytotoxic reactive oxygen species (ROS) are produced in the cells, which can inhibit the activity of intracellular proteins, break DNA chains and damage cell membranes containing large amounts of unsaturated fatty acids, causing various ROS can inhibit intracellular protein activity, break DNA strands and damage cell membranes containing large amounts of unsaturated fatty acids, thus causing various metabolic defects, aging, mutations and even cell death (Bauche and Laval, 1999; Lim et al., 2019).

Studies have shown that only 20% of the direct damage to DNA is caused by ionizing radiation, while the remaining 80% is indirectly caused by the attack of ROS generated by radiation. There are three main types of radiation-generated ROS: hydroxyl radicals (−OH), superoxide anion radicals (−O^2–^), and hydrogen peroxide (H_2_O_2_), which constitute strong oxidative damage to biomolecules such as proteins, lipids, nucleic acids, and sugars in cells (Bauche and Laval, 1999; Misra et al., 2012). Therefore, in addition to the efficient DNA damage repair system, the strong scavenging ability and extreme resistance to ROS by the antioxidant defense system in *D. radiodurans* is another important factor for its extreme radioresistance. The antioxidant protection system, overseen by *D. radiodurans*, primarily encompasses two categories of substances: the antioxidant enzyme system and non-enzymatic ROS scavengers. Within the antioxidant enzyme system, notable components are superoxide dismutase (SOD), catalase (CAT), and peroxidase (POD). The non-enzyme category comprises substances such as carotenoids, Mn2 + complexes, pyrroloquinoline quinone, and DNA protection during starvation (Dps) protein (Sadowska-Bartosz and Bartosz, 2023). Two Dps proteins, Dps1 (DR2263) and Dps2 (DRB0092), have been identified in *D. radiodurans*. The dimerization of the Dps1 protein protects DNA from hydroxyl radical damage (Grove and Wilkinson, 2005).

*Deinococcus radiodurans*, as a tough organism that can withstand extreme radiation and various adversities, represents the limit of survival of life against radiation and other adversities. Therefore, the study of *D. radiodurans* extreme radiation resistance mechanism has become a source of new ideas and methods in the fields of environmental protection and bioremediation, human health, food/drug research and development, plant and animal breeding and cosmetics, and even extraterrestrial space development and utilization, which has a very broad application prospect and important significance. *D. radiodurans* is an attractive candidate for genetic engineering applications due to its powerful DNA repair mechanism and efficient DNA uptake system (Rajpurohit and Misra, 2013).

The engineered strain *D. radiodurans* DRG300 was produced in a study in which the enhanced green fluorescent protein (eGFP) was fused with the promoter of the key DNA damage-inducible *recA* gene derived from *D. radiodurans* and transformed into the *D. radiodurans* R1 (Kota and Misra, 2006; Mishra et al., 2019; Rajpurohit et al., 2021). This strain serves as a potential whole-cell biosensor, enabling the construction of a detection system for real-time monitoring of radioactive and toxic pollutant hazards in the environment (GuanJun et al., 2008). A γ-radiation-inducible small RNA (sRNA), *DrsS*, was discovered in *D. radiodurans*, which is induced by oxidative (e.g., H_2_O_2_) and genotoxic stress. The research indicates that *DrsS* activates catalase and detoxifies intracellular reactive oxygen species (ROS) under oxidative stress (Kamble et al., 2010; Rajpurohit and Misra, 2010; Kota et al., 2014; Rai and Dutta, 2024). Recently, a research team successfully developed the recombinant strain *Deino-Ure* by genetically engineering *D. radiodurans* to express the urease gene (*Ure*) from *Sporosarcina pasteurii* DSM33. The urease in this strain hydrolyzes urea to produce CO_3_^2–^, which, in the presence of calcium ions, facilitates calcium carbonate precipitation, thereby enhancing *D. radiodurans*’ biomineralization and uranium immobilization capabilities (Liang et al., 2024). Furthermore, exosomes from *D. radiodurans* have been shown to substantially increase the survival rate of mice exposed to 8 Gy radiation (70%) while simultaneously safeguarding the hematopoietic and gastrointestinal systems. These exosomes are more effective than current single-organ radioprotectants and do not cause any toxicity (Han et al., 2025). Despite extensive research on the highly efficient DNA repair mechanisms, extreme antioxidant system and unique physicochemical properties of *D. radiodurans*, many genes, proteins and other biomolecules with unknown functions still exist (Bauche and Laval, 1999). TnpB from *D. radiodurans* ISDra2 is an RNA-directed nuclease that cleaves the 5′-TTGAT transposon-associated motifs next to DNA. TnpB was also identified as a progenitor of the CRISPR-Cas (Clustered Regularly Interspaced Short Palindromic Repeats) nuclease and used as a prototype for a new genome editing system (Karvelis et al., 2021).

## 2 Overview of the CRISPR/Cas system

In about half of the fine bacteria and almost all archaea, CRISPR and CRISPR-Cas associated proteins constitute an adaptive immune system against phage and exogenous plasmid invasion, called the CRISPR-Cas system (Jansen et al., 2002). This system was discovered in *E. coli* by Japanese scientists Ishino et al. (1987). The CRISPR/Cas system can recognize exogenous DNA and cut them off to silence the expression of exogenous genes. This is similar to the principle of RNA interference (RNAi) in eukaryotes. It is due to this precise targeting function that the CRISPR/Cas system has been developed as an efficient genome editing tool. Unlike genome editing that requires the assistance of Cre and I-SceI nucleases, CRISPR/Cas-based technologies do not require the pre-integration of unique enzyme recognition sequences into the genome, but instead use synthetic single-guide RNA-sgRNA (Jinek et al., 2012) (a chimera of crRNA and tracrRNA), or just crRNA, to direct the nucleic acid endonuclease Cas protein to target any site on the genome for genomic cleavage (Knott and Doudna, 2018). In recent years, transposon families and highly abundant OMEGA systems (including TnpB and IscB) have attracted widespread attention (Altae-Tran et al., 2021). These proteins are considered ancestors to Cas9 and Cas12, characterized by compact molecular sizes (approximately 400 amino acids) and diverse types, and have been demonstrated to possess RNA-guided nuclease activity (Altae-Tran et al., 2021; Karvelis et al., 2021). Notably, the Fanzor protein (a TnpB homologous protein widely present in eukaryotes) has also been shown to exhibit RNA-guided nuclease activity, indicating that RNA-guided nuclease systems are extensively distributed across trilobate organisms (Saito et al., 2023). In nature, CRISPR/Cas systems possess several classes, among which the CRISPR/Cas9 system is the most intensively studied and the most maturely used one.

### 2.1 CRISPR/Cas system classification

**FIGURE 1:**
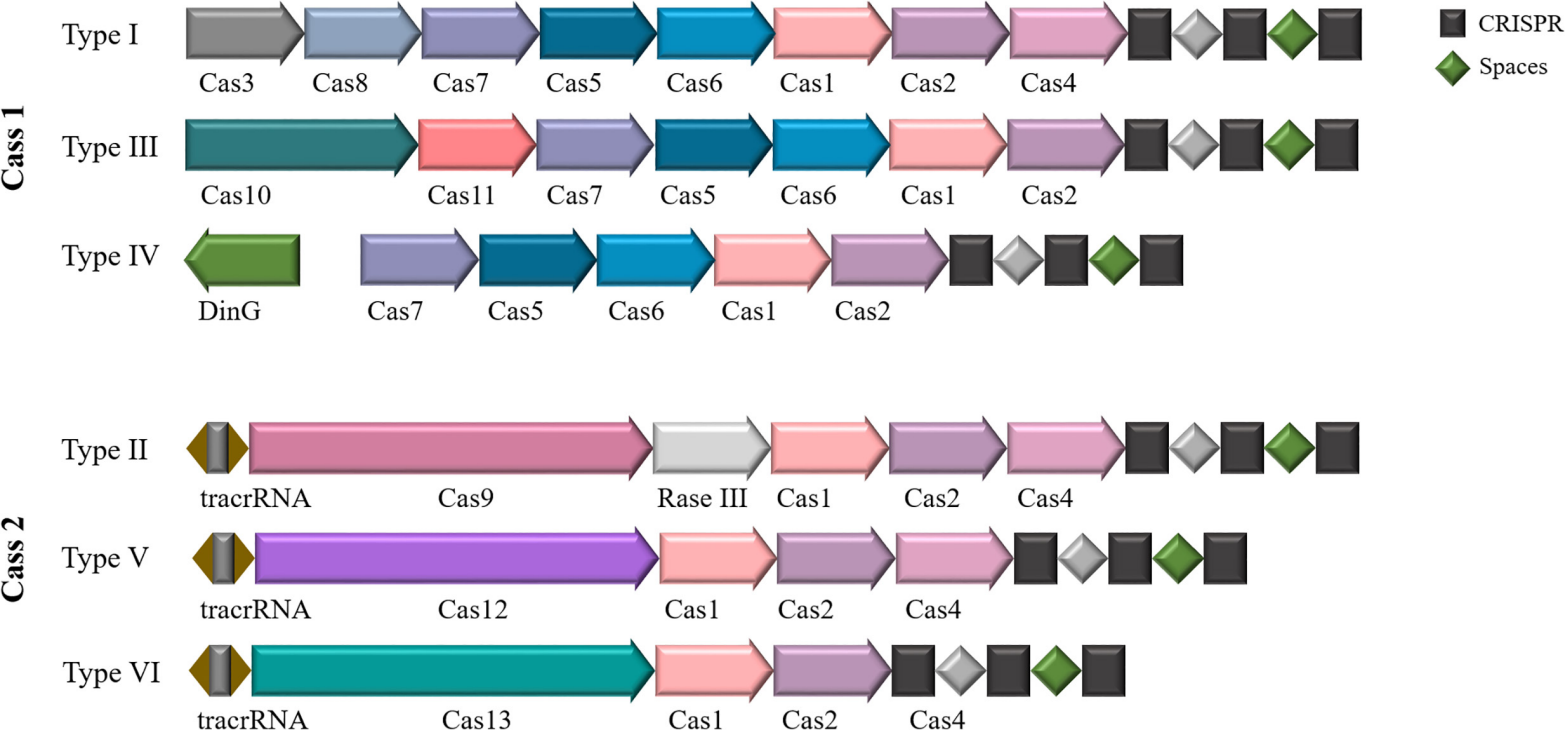
CRISPR-Cas system classification. Two classes and six types based on the structure and function of the Cas protein. Class I includes types I, III, and IV, and class II includes types II, V, and VI.

Depending on the structure and characteristics of the complex formed by the Cas effector protein during its function, CRISPR-Cas systems can be generally classified into two major classes (Haft et al., 2005; Makarova et al., 2011, 2020), including six types and 48 isoforms (Figure 1). These systems include three types: type I, type III, and type IV, represented by Cas3, Cas10, and Csf1, respectively. The type II, CRISPR-Cas, requires only a single Cas protein and performs immune functions by binding to crRNA to degrade exogenous invasive DNA. Approximately 90% of all CRISPR-Cas systems known to date fall into the first category. Class II CRISPR/Cas systems are mainly found in bacteria, with a relatively small distribution in archaea. Class II CRISPR-Cas systems also include three types: type II, type V and type VI, represented by Cas9, Cas12a (Cpf1), Cas13a (C2c2) and Cas12f (Cas14), respectively. Since this system relies on the formation of a complex between a single Cas nuclease and crRNA to function (Chylinski et al., 2014; O’Connell, 2019; Makarova et al., 2020), it is simple and efficient, so the understanding of its gene composition, mechanism of action and molecular structure of effectors is more thorough and clearer than that of type I system.

Current genome editing methods generally use Type II and Type V systems, using Cas9 and Cas12 as effector proteins. TnpB and IscB transposon proteins may be the ancestor proteins of Cas12 and Cas9, respectively (Kapitonov et al., 2015; Shmakov et al., 2015, 2017; Makarova et al., 2020). Notably, while CRISPR-like systems were previously thought to exist exclusively in prokaryotes, recent studies have identified homologous proteins of TnpB and IscB in eukaryotes, demonstrating their RNA-guided nuclease activity (Altae-Tran et al., 2021; Karvelis et al., 2021; Saito et al., 2023).

The CRISPR-Cas system is the most popular gene editing tool in the world today, but the size of conventional Cas nucleases is generally too large, limiting its application to mammals. In recent years, scientists have been seeking to develop smaller gene editing tools. Three research teams from China, Korea, and the United States have published research papers that identify the same class of ultra-small CRISPR nucleases, Cas12f (Wu et al., 2021; Xu et al., 2021; Kim et al., 2022). Cas12f is the smallest RNA-guided nuclease known to play a role in human cell editing and has been less studied compared to Cas9, Cas12 and Cas13. Bioinformatic analysis suggests that TnpB may be the predecessor of CRISPR-Cas9/Cas12 nucleases (Kapitonov et al., 2015; Shmakov et al., 2017; Makarova et al., 2020), and the nearest TnpB neighbor in the evolutionary tree is the miniature Cas12f nuclease. Therefore, a more detailed description of Cas12f nucleases is given below.

## 3 Overview of micro-CRISPR nuclease Cas12f and its application in the field of gene editing

### 3.1 CRISPR - Cas12f system structural features

In, Harrington et al. (2018) identified a CRISPR-Cas system containing Cas14 in uncultured archaea and found that it has similar structural domains to Cas9 and Cas12, but with functional differences. Sequence databases identified Cas12f as belonging to the class II CRISP V-F subtype of the R-Cas system, and a recent classification classified the family into Cas12f1 (Cas14a and V-U3 types), Cas12f2 (Cas14b) and Cas12f3 (Cas14c, V-U2 and U4 types) (Shmakov et al., 2017; Harrington et al., 2018; Yan et al., 2019; Makarova et al., 2020).

Cas12f nucleases share a common set of features (Jinek et al., 2012; Zetsche et al., 2015; Shmakov et al., 2017; Liu et al., 2019; Yan et al., 2019; Pausch et al., 2020): the inclusion of a RuvC nuclease structural domain similar to Cas12; preferential binding and cleavage of double-stranded DNA (dsDNA) at higher temperatures (45 °C–55 °C); and non-specific single-stranded DNA (ssDNA) degradation after target binding. However, compared to other typical class II nucleases, Cas12f is again distinctive in several properties: (i) Cas12f nuclease is relatively small, with a size of 400–700 amino acid residues, which is only half the size of other class II CRISPR effector nucleases (950–1400 aa), and is the smallest RNA-guided nuclease proven to date (Harrington et al., 2018; Savage, 2019). One of them, Cas12f1 (also known as Cas14a1), consists of 529 residues and lacks detectable sequence identity with other known proteins except for the presence of the RuvC structural domain. (ii) Cas12f1 is associated with a dual crRNA: tracrRNA guide (which can be combined into single guide RNA, sgRNA) and uses TTTR (where R is A or G) PAM (protospacer adjacent motifs) to cleave single- and double-stranded DNA targets (Jansen et al., 2002; Makarova et al., 2011; Harrington et al., 2018). The sequences of DNA cleavage products showed that Cas12f cleaves TS and NTS at 24 and 22 nucleotides upstream of PAM, respectively (Takeda et al., 2021). (iii) Dimerization after binding single-copy gRNA. (iv) CRISPR-Cas12f nuclease, in a PAM-dependent manner recognizes and cleaves dsDNA, whereas cutting ssDNA does not require the target sequence PAM. Cas12f recognizes T-rich or C-rich PAM (Bigelyte et al., 2021), Cas12a (Cpf1) recognizes T-rich PAM and Cas9 recognizes G-rich PAM for cleavage (Jinek et al., 2012). (v) Asymmetric double-stranded cleavage pattern. It was found that the mechanism of DNA cleavage by As-Cas12f1 differs greatly from previous Cas proteins (Wu et al., 2021). Previously reported Cas proteins usually cut two times inside the DNA targeting sequence, severing two strands of DNA and leaving a cut that is usually a neat flat end or a sticky end with only a few bases. In contrast, the core cut site of AsCas12f1 is outside of the target sequence, making one cut on the target strand and two cuts on the non-target strand, respectively, for a total of three cuts. This creates a set of asymmetric cuts that leave a longer stretch of sticky ends on the target strand. This asymmetric double-stranded cut pattern is currently rare. (vi) Cas14a shows high fidelity in the recognition of ssDNA substrates with targeting cleavage activity against ssDNA, as this recognition is mediated by the seed sequence near the middle of the ssDNA target interaction mediated, which is different from the seed region required to target the CRISPR-Cas12 system of dsDNA and PAM distal sequences, which are located in the middle of Cas14a (Wiedenheft et al., 2011; Chen et al., 2018; Aquino-Jarquin, 2019). (vii) The tracrRNA is very long. Despite the fact that Cas12f1 proteins are the most compact class II CRISPR-Cas nucleases (<500 aa) characterized so far, their gRNA length clearly exceeds the length of other class II effectors. These include crRNAs that are 40–50 nucleotides, component repeat sequences and spacer regions, and a long tracrRNA (153 and 169 nt for SpCas12f1 and AsCas12f1, respectively) (Wu et al., 2021). (viii) The guide RNA of Cas12f1 lacks sequence similarity to other Cas12 enzymes.

### 3.2 Mechanism of action of the CRISPR - Cas12f system

**FIGURE 2:**
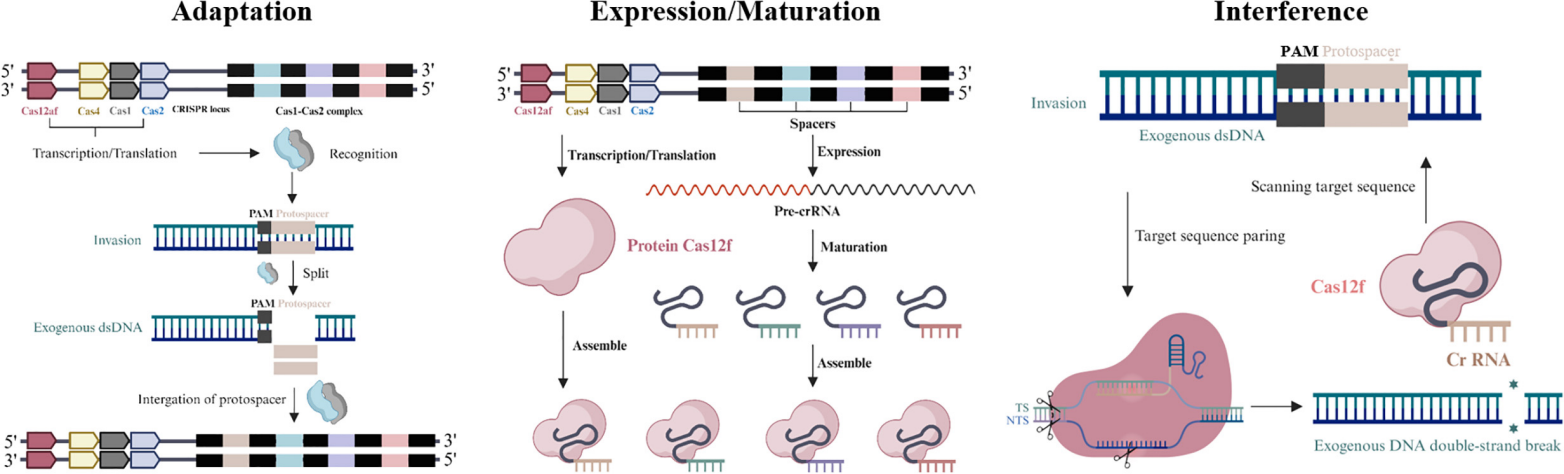
Stages of CRISPR-Cas12f system immunity: adaptation, expression/maturation, interference. Adaptation: the Cas protein complex like Cas1-Cas2 complex integrates fragments of invader DNA as spacers in the CRISPR array. Expression/Maturation: precursor CRISPR ribonucleic acid (pre-crRNA) is synthesized by transcription of pro-spacer sequences. Interference: the pre-crRNA is cleaved into short crRNAs and paired with tracrRNAs to form sgRNA by Cas proteins.

The defense process of the CRISPR-Cas12f system is similar to other CRISPR-Cas systems and consists of 3 phases: In the first phase, the Cas protein recognizes foreign nucleic acids, targets the pro-spacer sequences of the invading genome for cleavage, acquires new spacer sequences, and integrates them into the host genomic CRISPR array. In the second stage, these incorporated pro-spacer sequences are transcribed into precursor CRISPR ribonucleic acid (pre-crRNA). In the final stage, the pre-crRNA is cleaved into short crRNAs and paired with tracrRNAs to form guide RNA (sgRNA) by the action of Cas proteins. Cas12f requires dimerization of guide RNA, a catalytic effector complex that recognizes sgRNA and its target DNA to form the Cas12f-sgRNAtargetDNA complex, which targets the target nucleic acid region by crRNA and enables cleavage of exogenous nucleic acids to counteract reinvasion by homophages or exogenous DN A re-invasion (Mohanraju et al., 2016; Hille et al., 2018; Takeda et al., 2021; Figure 2). The asymmetric cleavage pattern of AsCas12f1 originates from its unique structural dynamics mechanism. AsCas12f1 (only 422 amino acids in size) first unwinds the DNA within the protospacer and creates a single-strand nick on the non-target strand (NTS) upon binding to target DNA. Subsequently, its bidirectional exonuclease activity extends the unwinding region to a 5-bp area beyond the protospacer, ultimately cleaving the target strand (TS) at a position 3 nucleotides downstream of the protospacer, generating three break sites and resulting in asymmetric cleavage (Song et al., 2024). This pattern differs from other Cas proteins. For instance, Cas9 typically makes two cuts within the DNA target sequence, cleaving both strands and leaving either blunt ends or sticky ends with only a few base overhangs. From evolutionary and functional perspectives, AsCas12f1’s asymmetric cleavage mode holds significant implications. While Cas12a exhibits more regular cleavage patterns that facilitate precise control over gene knockout and insertion in conventional gene editing, the unique termini generated by AsCas12f1’s asymmetric cleavage may be more conducive to ligation with specially designed DNA sequences, offering novel strategies for gene editing. Furthermore, studies have shown that Cas12f nucleases (including AsCas12f1) produce relatively fewer harmful byproducts such as chromosomal translocations and large fragment deletions during editing, making them particularly promising for applications requiring high genomic stability, such as gene therapy (Xin et al., 2022). Additionally, the small molecular size of AsCas12f1, combined with its cleavage pattern, enables easier packaging into adeno-associated virus (AAV) vectors for *in vivo* delivery, providing a superior tool for *in vivo* gene therapy and related applications.

### 3.3 CRISPR - Cas12f in the field of gene editing

To correct mammalian genetic defects, a set of gene editing tools must be delivered precisely to a specific tissue or cell, a process that typically uses adeno-associated viruses (AAVs), which do not make people sick and do not replicate themselves and are currently the most reliable vectors. The disadvantage of AAVs, however, is their limited loading capacity, and the current mainstream gene editing tools, dominated by Cas9 and Cas12a, generally exceed the loading limit of a single AAV. Cas12f nucleases are relatively small, half the size of other class II CRISPR effector nucleases, and hold promise for development as tools for mammalian cell editing.

Although natural Cas12f and its sgRNA are inactive in mammalian cells, Kim et al. (2022) have modified the system to greatly enhance the gene editing activity of the Un1Cas12f1 system in mammals. Based on the structural information of the Un1Cas12f1/sgRNA/DNA complex, truncating the RNA portion of the structure defined as disordered, while adding uridylic acid-rich sequences at the end of the crRNA, the engineered Un1Cas12f1 system showed efficiency comparable to that of SpCas9 and specificity similar to that of AsCas12a. At the same time, the system’s volume of only 529 aa allows Un1Cas12f1 to be efficiently delivered via AAV, which meets the volume requirements of AAV viral delivery systems (Kim et al., 2022). The high gene editing efficiency and specific genome editing ability of Cas12f in mammalian cells have revealed its great potential for clinical therapeutic applications.

Wu et al. (2021) characterized the DNA recognition and cleavage mechanism of AsCas12f1, a very small CRISPR nuclease. Cas12f1 can effectively execute gene editing in *E. coli* and *Klebsiella pneumoniae*, as well as a variety of human cells, through plasmids, ribonucleoprotein (RNP) and AAV. This discovery offers a new idea for the development of miniature precision gene editing and therapeutic tools. Wang et al. (2021) successfully demonstrated genome editing in *Bacillus anthracis* based on the AsCas12f1 nuclease from *Acidibacillus sulfuroxidans*. Additionally, they constructed a dual plasmid CRISPR-AsCas12f1 system. The results indicated that the CRISPR-Cas12f system is a highly effective tool for genetic manipulation in *Bacillus anthracis* and may be broadly applicable to other *Bacillus* strains. The CRISPR-Cas12f system has a clear comparative advantage over the CRISPR-Cas9 system, as it has smaller genome editing plasmid size and higher transformation efficiency in *B. anthracis* related experiments using this effector. Bigelyte et al. (2021) demonstrated that two V-FCRISPR-Cas nucleases, SpCas12f1 (497 aa) and AsCas12f1 (422 aa), are capable of targeted DNA modification in both plant and human cells. This discovery paves the way for the development of miniature Cas12f1-based genome editing tools. At the same time, the development of diagnostics based on the CRISPR-Cas12f system, as well as the design and modification of these systems, has enabled the development of a variety of rapid assays. Cas14a binds more specifically to its targets than Cas12a, making it better suited for the development of assays that require single-base resolution. Harrington et al. (2018) used phosphorothioate amplification to protect a strand from exonuclease degradation, leaving behind ssDNA substrates that Cas14a can detect. They developed the Cas14a-DETECTR assay, which has high fidelity in distinguishing between ssDNA substrates and can be used for DNA single nucleotide polymorphism (SNP) genotyping, ssDNA pathogen detection, and diagnosis. The team successfully used Cas14a - DETECTR to detect SNP typing of the human E3 ubiquitin ligase (HERC2) gene, which contains SNP typing that leads to iris color variation. The team designed the Cas14-DETECTR assay, which can successfully differentiate between blue and brown eye genotypes with a single base of specificity (only one base difference in the sequence of the genes controlling the two eye colors) (Harrington et al., 2018). Meanwhile, the Cas14- DETECTR platform can also be used as a viral screening method. The CRISPR-Cas14 system is used in conjunction with the HUDSON method (Gootenberg et al., 2017), which does not involve complex sample extraction and transportation, to achieve rapid detection of viruses, and has been successfully applied to the fine virus detection of human bocavirus (HBoV1) (Aquino-Jarquin, 2019).

Wei et al. (2021) developed a highly sensitive and specific fluorescent tool for bacterial detection using Cas14a1-mediated nucleic acid detection platform (CMP). Combining CMP with molecular amplification to construct a CRISPR-Cas-based bioanalytical technology allows for rapid nucleic acid detection with high sensitivity and specificity (Wei et al., 2021). The results show that CMP is a reliable and accurate pathogen detection method. The technique can accurately identify different types of pathogens in milk samples. Wu et al. (2022) developed a novel universal Cas14 -pMOFs fluorescent sensor by combining the CRISPR- Cas14a system with two-dimensional porphyrin metal-organic skeleton nanosheets. Using an aptamer to introduce a homogeneous competitive reaction between Microcystin-LR (MC-LR) and complementary DNA, this novel sensor was applied to sensitively determine MC-LR with a limit of detection of 19 pg/mL (Ma et al., 2014; Yang et al., 2020; Wu et al., 2022). The Cas14- pMOFs fluorescence sensor was shown to be feasible and applicable as a favorable MC-LR assay, and the application of CRISPR-Cas14a-based assays for non-nucleic acid target detection was extended.

**FIGURE 3:**
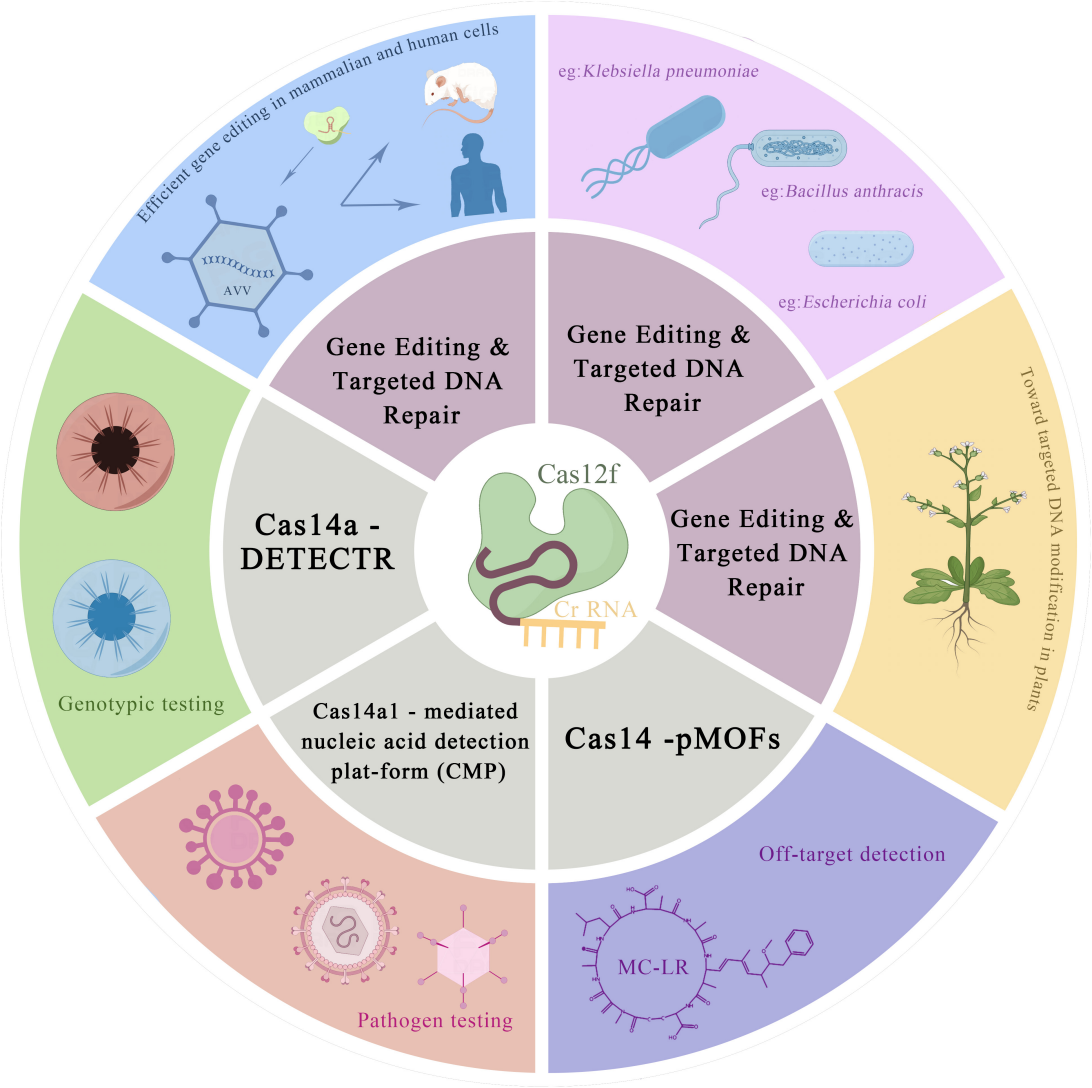
A general overview of application of CRISPR-Cas12f in the field of gene editing.

However, it is also important to recognize that while CRISPR-Cas12f is an effective and cost-effective method for high-throughput pathogenic mutation screening, sgRNA-mediated Cas12f targets the cleavage of ssDNA, and additional steps are required to generate ssDNA from dsDNA targets prior to detection, and this particular property of CRISPR-Cas12f may increase the complexity of its application (Figure 3).

## 4 The evolutionary relationship between CRISPR-Cas and TnpB

Over the past decade, scientists have applied the CRISPR-Cas9 system, a precisely programmable system for modifying DNA, to gene editing technology. In addition to generating DNA double-strand breaks, deactivated Cas (dCas) or Cas nickase (nCas) versions have been designed as reprogrammable DNA binding or cleavage systems. Different domains have been fused to dCas or nCas to establish epigenome editing, base editing and primitive editing technologies. The origin of CRISPR-Cas enzymes has also been a hot topic of research in recent years, and researchers have made new discoveries on the subject, as they have identified a new programmable DNA modification system. The TnpB system consists of nuclease and ωRNA components. The ωRNA comprises a scaffold region forming complex three-dimensional structures and a guide region that complements the target DNA. While the OMEGA system contains small nuclease molecules, the ωRNA in TnpB is typically longer and more structurally complex compared to crRNA (Kato et al., 2022; Schuler et al., 2022; Nakagawa et al., 2023; Saito et al., 2023; Sasnauskas et al., 2023). The TnpB ωRNA measures approximately 150 nt, consisting of a 116 nt scaffold region and a 16 nt guide region. It features four stems and one pseudoknot (a circular sequence in the stem-loop structure that pairs with base sequences elsewhere, forming a “knot-like” shape), which is also preserved in all Cas12 systems (Nakagawa et al., 2023; Sasnauskas et al., 2023). However, TnpB differs significantly from Cas12 systems. Structural comparisons reveal that certain regions of the TnpB ωRNA function similarly to specific domains of the Cas12 protein (Nakagawa et al., 2023; Sasnauskas et al., 2023). Furthermore, a single ISDra2 TnpB protein is guided by a single ωRNA, while certain Cas12 proteins form asymmetric dimers (e.g., Cas12f) that require dual RNA guidance (tracrRNA and crRNA) (Takeda et al., 2021; Xiao et al., 2021). Structural analysis suggests that during evolution, Cas12 likely identified PAM-terminal guide sequences through either forming asymmetric dimers or inserting diversified REC2 domains, thereby establishing the structural basis for CRISPR-Cas adaptation (Nakagawa et al., 2023). The dual-RNA guidance sequence may have evolved from ωRNA. Cas12f1 and dual crRNA: tracrRNA-guided (capable of forming sgRNA) using TTTR (where R is A or G) PAM to cleave single-stranded and double-stranded DNA target (Harrington et al., 2018; Karvelis et al., 2020; Takeda et al., 2021). Cas12f requires dimerization of the guiding RNA. This catalytic effector complex recognizes sgRNA and target DNA, forming Cas12f-sgRNA-targetDNA complexes (Takeda et al., 2021). Through crRNA targeting of nucleic acid regions, it achieves exogenous DNA cleavage to combat re-invasion by phage or foreign DNA (Mohanraju et al., 2016; Hille et al., 2018; Takeda et al., 2021).

### 4.1 The widespread IS200/605 transposon family encodes TnpA and TnpB

Transposition plays a key role in remodeling the genomes of all organisms. Insertion sequences of the IS200/IS605 and IS607 families are among the simplest mobile genetic elements, containing only the genes required for their transposition and their regulation (Siguier et al., 2014; Koonin, 2016). These elements encode tnpA transposases, which are essential for mobilization, and usually carry an accessory tnpB gene, which is dispensable for transposition. Although the role of TnpA in IS200/IS605 transposon mobilization is well documented, the function of TnpB remains largely unknown. Three independent experiments on IS607, ISHp608 and ISDra2 elements (the latter two belonging to the IS200/IS605 family) have shown that TnpB is essential for transposition in *E. coli* and *D. radiodurans* (Ishino et al., 1987; Kersulyte et al., 2000, 2002). Interestingly, many IS elements (e.g., IS1341, IS809 and IS1136) encode TnpB as the sole protein (putative transposase), but supporting evidence for TnpB-mediated transposition is still lacking. As with other elements of the IS200/IS605 and IS607 families, these TnpB-only transposons lack terminal inverted repeat sequences (TIR) and target site replication (TSD). Studies have shown that prokaryotic TnpB proteins are encoded by bacterial transposons of the IS200/605 or IS607 families. TnpB-like proteins (Fanzor1 and Fanzor2) are found in a wide range of eukaryotic transposable elements (TES) and large dsDNA viruses that infect eukaryotic cells (Bao and Jurka, 2013). The simplest and oldest mobile genetic elements (MGEs) are insertion sequences from the IS200/IS605 and IS607 families. Typically, encode TnpA and TnpB genes, or isolated TnpA or TnpB genes, in various configurations and contain subterminal left-end (LE) and right-end (RE) palindromic elements at the end of the MGE (ISfinder database).

### 4.2 TnpB protein is the ancestor of Cas12 nuclease

The fully characterized *D. radiodurans* ISDra2 of the IS200/IS605 family consists of partially overlapping TnpA and TnpB genes flanked by LE and RE elements (May and Craig, 1996; Bernales et al., 1999; Islam et al., 2003; Hickman et al., 2010; Pasternak et al., 2010; Koonin, 2016). For a given IS605 transposon element, the TnpA protein binds to subterminal palindromic sequences located at the left and right ends (LE/RE) of the element, and catalyzes the cleavage and rejoining of DNA substrates through a “Peel and Paste” mechanism (He et al., 2015). During this process, TnpA recognizes a cleavage sequence positioned upstream of the LE (referred to as CL, spanning 4–5 nucleotides), which also serves as the target site (TS) for transposon insertion. In studies examining the editing activity of TnpB derived from ISDra2 (a member of the IS605 family), the aforementioned research proposed a “Peel, Paste and Copy” model (Karvelis et al., 2021). This model involves three key steps: (1) TnpA facilitates the excision of the transposon from its original genomic location and its subsequent insertion into a new site; (2) guided by right-end element RNA (reRNA), TnpB identifies the transposon-associated motif (TAM)–which appears to match the CL sequence–and induces a double-strand break at the transposon’s original position; (3) the resulting break is repaired via recombination with the allele containing the transposon. This function of TnpB as a homing endonuclease allows for the direct prediction of TnpB protein, reRNA, and TAM sequences from genomic data. Such predictability would pave the way for large-scale screening of TnpB as a compact genome-editing tool (Xiang et al., 2024). *D. radiodurans* ISDra2 TnpA is a very small Y1 transposase (140 aa) of the HUH family that excises a specific DNA strand near the 5′-TTGAT sequence to form a single-stranded transposon loop, which is then integrated 3′ into a new position in the TTGAT target to complete the transposition cycle without repeating the target site (Siguier et al., 2006; Pasternak et al., 2010).

Recent research has elucidated the evolutionary pathways of the CRISPR-Cas system, indicating that the prokaryotic transposon-encoded IscB and TnpB proteins may serve as ancestors of the Cas9 and Cas12 nucleases, respectively (Altae-Tran et al., 2021; Karvelis et al., 2021). These ancestors exhibited a small size, yet there was insufficient evidence for their nuclease activity was lacking; it was not until 2021 that IscB and TnpB were found to cleave dsDNA under the guidance of non-coding RNAs (ωRNA or reRNA), thereby confirming a similar working mechanism to the CRISPR-Cas system (Altae-Tran et al., 2021; Karvelis et al., 2021). TnpB is encoded by the IS200/IS605 and other prokaryotic transposon family and is presumed to be involved in transposon expansion. TnpB is widely distributed, with over a million copies present in the currently known genome. Nevertheless, previous studies have identified only one TnpB nuclease (ISDra2) that is active in human cells and inefficient. Additionally, TnpB targets key DNA-dependent cleavage components (e.g., reRNA) that may be associated with transposons and hence can be predicted based on transposon information, which would facilitate the development of tools.

OMEGA (obligate mobile element–guided activity) was discovered by researchers within the IscB protein family, which is believed to be the ancestors of CRISPR-Cas9 enzymes (Altae-Tran et al., 2021). Both IscB and TnpB belong to the IS200/IS605 superfamily transposon-encoding proteins. Among them, IscB is considered as the ancestor of Cas9, while TnpB is the ancestor of Cas12. They are collectively referred to as OMEGA proteins, which guide RNA called ωRNA, revealing their deep evolutionary association with transposons (Shmakov et al., 2017; Altae-Tran et al., 2021). Structurally, IscB contains RuvC and HNH nuclease domains homologous to Cas9, while TnpB possesses the Cas12 family-conserved RuvC domain. These core structures form the basis of their RNA-guided nuclease activity. Their ωRNA contains complex structures such as pseudoknots and stem loops, which are considered evolutionary precursors to crRNA and tracrRNA in CRISPR-Cas systems (Nakagawa et al., 2023; Sasnauskas et al., 2023). Additionally, OMEGA systems are widely distributed among trilobate organisms, not only abundant in prokaryotes but also present in eukaryotic proteins like Fanzor (homolog of TnpB) that exhibit RNA-guided nuclease activity (Altae-Tran et al., 2021; Sutandy et al., 2023; Burstein et al., 2017). This indicates that RNA-guided nuclease activity is an ancient and conserved feature initially serving selfish purposes like transposon retention and dissemination. Subsequently inherited by CRISPR-Cas systems, it has been adapted into immune tools against exogenous DNA. This evolution clearly demonstrates the evolutionary origin pathway of IscB and TnpB as RNA-guided nuclease systems. Systematic phylogenetic analysis reveals that all Cas9 proteins share a common evolutionary origin within the IscB lineage. Altae-Tran et al. (2021) proposed that IsrB likely evolved from a compact RuvC nuclease, later acquiring the HNH domain (possibly through insertions of IshB-like proteins) to form the IscB family. Structural characterization further supports these conclusion (Kato et al., 2022; Schuler et al., 2022). Further research is needed to clarify TnpB’s evolutionary relationship with IscB and its origin from RuvC nuclease.

In addition the several IscB and IsrB families, most IS200/IS605 transposons encode RuvC-like endonucleases from another family, TnpB, which is thought to be the ancestor of the V-type CRISPR effector Cas12s (Shmakov et al., 2017; Altae-Tran et al., 2021). TnpB may also be the ancestor of the bigger protein Fanzors, which are encoded in different eukaryotic transposons (Pasternak et al., 2010). The TnpB family, including Fanzor, is significantly more diverse than the IscB family; an HMMER search in publicly available prokaryotic genomes identified more than one million TnpB motifs. Conserved non-coding regions were found downstream of the CDS of many TnpBs, indicating the presence of associated ncRNAs that could serve as RNA guides (Altae-Tran et al., 2021). These TnpB families are considered to be the ancestor of CRISPR-Cas12 enzymes.

However, there are important differences between TnpB and Cas12f nucleases. First, Cas12f nuclease uses gRNA from the CRISPR array, whereas TnpB uses right transposon element-derived reRNA as a guide. Second, TnpB is a monomer and requires a single reRNA molecule, whereas Cas12f nuclease is a dimer and binds to the crRNA (CRISPR RNA)-tracrRNA (trans-activating crRNA) duplex (Bao et al., 2009; Cock et al., 2010). Target Adjacent Motif (TAM) and Protospacer Adjacent Motif (PAM) are critical components in genome editing tools that enable nuclease recognition of target DNA. Their core function is to ensure nuclease specificity in identifying and cleaving target sequences, directly impacting editing efficiency, accuracy, and target coverage. These elements form the foundation for effective genome editing. Finally, although the TAM (transposon-associated motif) sequence required for TnpB cleavage appears to be equivalent to the PAM sequence that licenses Cas12f cleavage, Cas12f proteins show a different PAM requirement (Bernales et al., 1999). However, the evolutionary relationship between Cas12 nucleases and the IS200/IS605/IS607 family of MGES reflected in the extent to which the PAM diversity of Cas12f nucleases correlates with the different TAM sequence requirements of TnpB proteins. The main characteristics of TnpB, Cas12f and Cas9 are summarized in Table 1.

**TABLE 1:** Comparative characteristics of TnpB, Cas12f and Cas9.

Characteristics	TnpB	Cas12f	Cas9
Structural domain	N-terminal fragment (REC), C-terminal fragment (NUC)	RuvC	HNH, RuvC
Protein size (amino acids, aa)	369–408 aa (ISDra2 TnpB, 408 aa; ISYmu1, 369 aa)	400–700 aa (Cas12f1, 422 aa)	1,000–1,400 aa (SpCas9, 1368 aa)
Guide RNA type	ωRNA, reRNA	tracrRNA, crRNA	tracrRNA, crRNA
PAM/TAM requirement	TAM (ISDra2 recognizes 5′-TTGAT-3′, ISAam1 recognizes 5′-TTYAA-3′)	PAM (commonly identified as 5′-TTTN-3′)	PAM (5′-NGG-3′)
Cleavage pattern	5′ overhang	5′ overhang (three cuts)	Blunt end
Collateral activity	Yes/no	Yes	No
Host application	Prokaryotes, plants, mammalian cells.	Prokaryotes, eukaryotes, mammalian cells.	Prokaryotes, eukaryotes, plants and mammalian cells.

The TAM of TnpB is closely associated with the insertion sequence of transposons, resulting in its target selection being limited to specific TAM sequences. For example, TnpB from radiation-resistant *Pseudomonas aeruginosa* only recognizes the 5′-TTGAT-3′ TAM, while the archaeal SisTnpB1 specifically targets TTTAA. Although this high specificity reduces off-target risks, it significantly narrows the range of editable targets, particularly when genomic regions lacking corresponding TAM are unavailable. In contrast, Cas12f’s PAM-dependent mechanism is more flexible and can be engineered for expansion. For instance, while natural Cas12f primarily recognizes T-rich PAMs, the engineered CnCas12f1 can recognize C-rich PAMs, and Cas12Pro (modified from Cas12n) even identifies 5′-ANN PAMs, achieving 80% insertion/inducement efficiency in human cells (Chen et al., 2023). This modifiability allows broader genomic coverage and wider target selection. Reported TAM recognition sequences of TnpB from different microbial sources vary by approximately 5–6 bases, making existing TnpB protein recognition windows smaller compared to Cas9’s PAM recognition motifs (Sasnauskas et al., 2023; Xiang et al., 2024). Both IscB and TnpB utilize single non-coding RNAs for RNA-guided double-strand DNA cleavage, demonstrating genome-editing activity in human cells similar to the CRISPR/Cas genome editing system. However, the IscB system operates by targeting cleavage at 3 nt upstream of the TAM sequence, mirroring the CRISPR/Cas9 mechanism. In contrast, the non-target strand cleavage occurs at 8 or 12 nt upstream of the TAM, ultimately producing 5′ overhangs of 5 nt or 9 nt length. This characteristic differs from Cas9’s blunt-end cleavage while aligning with the Cas12 protein’s cutting patter (Altae-Tran et al., 2021).

### 4.3 Action mechanism of TnpB

TnpB, the ancestor of CRISPR-Cas nucleases that is a reprogrammable RNA-directed functional nuclease. It is a TAM-dependent RNA-guided dsDNA nuclease, guided by the similarity of TnpB to the CRISPR-Cas12f effector complex that acts as an RNA-guided dsDNA nuclease (Bernales et al., 1999). It has been proposed that the approximately 16-nt 3′ ends of reRNA may act as guide sequences to orient TnpB to its target and activate DNA cleavage. These ends are derived from DNA in the vicinity of the transposon, which is itself variable. The TTGAT sequence of the TnpB cleavage plasmid corresponds to the target region required for TnpA-mediated ISDra2 transposon excision and insertion. This region, known as TAM, is equivalent to the PAM sequence required by Cas9 or Cas12 nucleases to trigger DNA excision (Tavakoli and Derbyshire, 2001). TnpB cleaves the donor junction generated after transposon excision and proposes that TnpB-mediated DSB triggers homology-directed repair to transposon back to its original position (Rajpurohit et al., 2016). This process would be similar to the homing of group I introns facilitated by intron encoding nucleic acid endonucleases (Yang and Bennetzen, 2009). There, in MGES that contain both tnpA and tnpB, two types of transposition would be possible: (i) excision of the transposon and its insertion into a new site (catalyzed by TnpA); (ii) transposon “homing,” i.e., which is the process by which TnpB cleaves the DNA in the non-transposon allele, triggering the demonstration of the RNA-guided dsDNA cleavage activity of TnpB. This direct experimental confirmation of the evolutionary scenario of class 2 CRISPR-CAS systems identifies MGES as the predecessor of Cas9 and Cas12 effectors (Robertson, 1981; Adams et al., 1997; Smith and Thorpe, 2002). A comparison of the sequences of TnpB and Cas12 family proteins revealed that they share a similar structural domain organization, which includes conserved RuvC endonuclease-like motifs. The miniature Cas12f nuclease is the closest TnpB neighbor on the evolutionary tree (Robertson, 1981; Bernales et al., 1999; Roberts et al., 2015). The typical TAM sequence for ISDra2 TnpB is 5′-TTGAT-3′. However, unlike the strict constraints imposed by CRISPR’s PAM requirements, TnpB’s TAM sequence demands exhibit greater flexibility in genome editing. The PAM sequences in CRISPR-Cas systems are highly regulated, with the most widely used *Streptococcus pyogenes* Cas9 requiring two G nucleotides as its PAM sequence – a requirement that significantly limits the number of target sites it can accommodate. In contrast, TnpB demonstrates relatively flexible TAM sequence requirements. A study conducted by Professor Qunxin She’s research team at Shandong University on April 24, 2024 revealed that thermophilic archaeal TnpB can recognize diverse TAM variant sequences and mediate gene editing, including many non-casogenic TAM variants. The research also demonstrated that TnpB exhibits exceptional targeting specificity at weak TAM sites. Leveraging this property, scientists successfully achieved efficient single-nucleotide editing through TnpB-based homologous recombination (Feng et al., 2024). In addition, the research of Professor Nan Peng’s group at Huazhong Agricultural University also pointed out that SisTnpB1 only needs a non-conserved TAM sequence (5′-WNHNN-3′) to induce the cleavage of target DNA (Xu et al., 2023). Current strategies to overcome the limitations of TAM sequences primarily leverage TnpB’s recognition of weak TAM sequences. The use of diverse weak TAM sequences not only enables more flexible gene editing and improved cell survival rates, but also significantly expands the targeting scope. This approach may prove applicable to various CRISPR-Cas systems.

### 4.4 Applications of the TnpB protein

In the past 5 years, with the rapid development of gene editing technology, TnpB has shown significant potential in gene therapy, disease modeling and microbial engineering, and has become a new generation of miniature gene editing tools after the CRISPR-Cas system.

Altae-Tran et al. (2021) demonstrated that three different transposon-encoded proteins, IscB, IsrB, and TnpB, are naturally occurring, reprogrammable RNA-guided DNA nucleases. TnpB was first demonstrated to possess *in vitro* and *in vivo* cleavage activity for application as a genome editing tool in mammalian gene therapy (Altae-Tran et al., 2021). Experiments have demonstrated that TnpB editing in *E. coli* can efficiently cleave plasmids, as well as work in human cells. Karvelis et al. (2021) demonstrated that TnpB of *D. radiodurans* ISDra2 is an RNA-directed nuclease, which is induced by RNA, with a right-terminal element of the transposon that cleaves DNA next to the 5′-TTGAT transpo AAV-mediated son-related motif. TnpB can be reprogrammed to cleave DNA target sites in human cells and improves the efficiency and specificity by predicting RNA sequences using deep learning. Xiang et al. (2024) screened 78 TnpB systems and identified 33 active ones, including 5 that function in human cells. Notably, the ISAam1 and ISYmu1 TnpB outperformed three previously published Cas12f editors (e.g., Un1Cas12f1) in performance, demonstrating SaCas9-like capabilities while optimizing Nme2Cas9’s editing efficiency. However, TnpB transposase systems have not yet been applied to plant gene editing (Xiang et al., 2024). Li Q. et al. (2024) subsequently demonstrated the successful implementation of TnpB-mediated plant gene editing, revealing that the ISDra2 and ISYmu1 nuclease systems exhibit remarkably high efficiency. Notably, as TnpB nuclease specifically recognizes transactylated (TA)-rich target sequences in transacting actin (TAM), the TnpB system should complement the Cas9 system, which targets the adjacent NGG motif in the original spacer region containing guanine (G). This study establishes a foundation for developing alternative plant genome editing tools (Li Q. et al., 2024). The variants of TnpB with distinct activities (e.g., ISDra2, ISAam1, and ISYmu1) are proved in Table 2.

**TABLE 2:** The variants of TnpB with distinct activities.

TnpB variant	Source	Protein size (aa)	Editing efficiency/characteristics	TAM characteristics	References
ISDra2 TnpB	*Deinococcus radiodurans*	408	– Low efficiency of dsDNA cutting *in vitro*. – Requiring long crRNA (about 170 nt). – The first identified TnpB protase, highly efficient in bacteria (>80% target efficiency), and 30%–50% editing efficiency in human cells.	Identifing the TAM sequence 5′-TTGAT 3′ which must be present upstream of the target sequence.	Karvelis et al., 2021
ISAam1 TnpB	*Anoxybacillus amylolyticus*	369	– The editing efficiency is lower than ISDra2. – The cutting window is located 18–23 bp downstream of PAM. – It supports simplified design of sgRNA (about 60 nt).	Identifing the TAM sequence 5′-TTTAA 3′.	Badon et al., 2024
ISYmu1 TnpB	*Youngiibacter multivorans*	382	– Editable but lower than the ISDra2 variant in human cells. – The smallest natural TnpB. – Plant cell editing efficiency reaches 20%–30% – Off-target rate is significantly lower than Cas9. – Showing DNA cutting activity but low editing efficiency.	The recognition of the 5′-TTGAT 3′ TAM sequence was consistent with that of the ISDra2 TAM recognition sequence.	Badon et al., 2024

Sasnauskas et al. (2023) and Nakagawa et al. (2023), on the other hand, analyzed and demonstrated the cryo-electron microscopy structures of the TnpB protein-bound ωRNA complex from *D. radiodurans* in DNA-bound and free forms, respectively. They discovered that the ωRNA has a pseudo-knot structure and that the pseudo-knot in the sgRNA of the Cas12 protein is conserved (Nakagawa et al., 2023; Sasnauskas et al., 2023). These profiles of the protein’s physical structure suggest that TnpB is the minimal structural and functional core of the Cas12 protein family. This framework enables the development of TnpB-based genome editing tools and enhances our comprehension of the evolution from transposon-encoded TnpB proteins to CRISPR-Cas12 effectors (Sasnauskas et al., 2023; Marquart et al., 2024).

Given previous experience optimizing Cas proteins and gRNAs, TnpB activity could be improved while addressing TAM limitations. The recent discovery of the structure of the TnpB-reRNA-DNA complex will hasten this process. Researchers have modified TnpB to enhance its interaction with DNA, thereby increasing the efficiency of gene editing. A deep learning model was developed to predict the efficiency of TnpB targeting, which increased the editing activity by 4.4-fold, and the editing efficiency of TnpB in the mouse brain reached 65.9% (Marquart et al., 2024). Wang et al. (2024) utilized ISDra2-TnpB for gene editing in mice and validated the method *in vivo* in an *in vivo*-based AAV system for genome editing. Furthermore, the study successfully developed a truncated ultra-compact TnpB editor (only 379 aa in size) with a shortened CTD structural domain, which is smaller in size but retains efficient gene editing capabilities in mammalian cells and mice. The extremely compact non-Cas nuclease TnpB and the truncated ultra-mini ISDra2-TnpB can serve as very promising multifunctional tools that show great potential for a variety of genome editing applications (Wang et al., 2024). Li Q. et al. (2024) improved the nuclease activity of TnpB by stepwise modification of non-coding RNAs (ωRNAs or reRNAs) of the ISDra2 TnpB system. Modified TnpB-ωRNA system can be efficiently delivered *in vivo* with individual AAV and correct the disease phenotype in a mouse model of tyrosinemia, demonstrating its applicability for *in vivo* genome editing. In addition, against the Klkb1 gene target of hereditary angioedema (HAE), the TnpB-ωRNA system also showed high specificity for adult editing (Li Q. et al., 2024). It predicts the potential of miniature TnpB editing tools in the treatment of hereditary diseases, such as high cholesterol genetic defects, through gene editing. Chen et al. (2023) found, by evolutionary and biochemical analyses, that the miniature Cas12n nuclease (400–700 aa) was similar to the progenitor of the V-type CRISPR system - the TnpB, the transposition-associated nuclease, which may be the earliest evolutionary intermediate for TnpB to evolve into other V-type Cas nucleases. Cas12n nuclease recognizes a specific 5′-AAN PAM sequence, in which the A nucleotide at the -2 position is also required for TnpB. In addition, a highly effective CRISPR-Cas12n (called Cas12Pro) system with up to 80% insertion or deletion efficiency was developed for efficient base editing in human cells (Chen et al., 2023).

The above findings demonstrate that TnpB proteins with smaller molecular structures, which can be more easily delivered via viral vectors (e.g., AAV), are particularly important for practical applications in gene therapy. And it can be used as a powerful gene editing tool for precise gene editing in bacteria.

While the TnpB system (e.g., ISDra2-TnpB) has demonstrated high targeting efficiency in multiple studies, its off-target risks still require systematic evaluation. For instance, Wang et al.’s (2024) research further confirmed the significant potential of ISDra2-TnpB as an efficient tool for achieving site-specific modifications *in vitro* and *in vivo*. Their groundbreaking study using ISDra2-TnpB to generate mutant mice demonstrated its utility in genome editing based on *in vivo* adeno-associated virus (AAV). Additionally, the research introduced a truncated ultra-small TnpB editor (<400 aa) generated by shortening the C-terminal domain (CTD) of TnpB. This engineered TnpB editor achieved effective gene editing in mammalian cells and mice, with a 9.5% editing efficiency for the target gene PCSK9 and no detectable off-target effects verified through whole-genome sequencing (Wang et al., 2024). The modified TnpB editor not only maintained high editing efficiency but also enhanced AAV payload flexibility. The University of Zurich team optimized the design of TnpB from *D. radiodurans* (ISDra2), resulting in TnpBmax for mammalian cells that achieved an average 4.4-fold improvement in editing efficiency. They also developed variants with mutations at the K76 site, which can recognize adjacent motifs (TAM) of alternative targets, thereby expanding the targeting range of ISDra2 TnpB. Through AI model predictions on 10,211 target sites, the optimized variant showed 75.3% efficiency in mouse liver, though some natural variants still carry non-specific cleavage risks. In a 2024 study by Xu Chunlong’s team, engineered ωRNA significantly enhanced TnpB’s targeting in tyrosinemia mouse models, though further RNA design optimization is needed to reduce off-target effects (Li Q. et al., 2024). While TnpB’s size advantages have been fully validated, clinical applications still face challenges in specificity, delivery efficiency, and safety. Future optimizations will focus on improving editing accuracy and delivery efficiency. AI-assisted design could develop highly specific and off-target-resistant variants, while engineered ωRNA structures could further enhance targeting recognition. Additionally, developing TnpB-based base editing tools could reduce DNA double-strand break risks, paving the way for safer gene therapies. The coordinated development of these technical approaches will drive practical clinical applications of TnpB systems.

### 4.5 Current limitations and future challenges of the TnpB system

Although TnpB, as a supercompact RNA-guided nuclease (approximately 400 amino acids), has demonstrated significant potential in gene editing applications such as AAV delivery compatibility and efficient eukaryotic editing, it still faces several critical limitations in widespread adoption that require further optimization and exploration. While proof-of-concept studies have been conducted in mammalian cells regarding editing efficiency, its overall performance remains inferior to CRISPR-Cas nuclease systems. The varying cleavage efficiencies across species also restrict its application in scenarios demanding high editing precision. Currently, TnpB is primarily used for gene knockout (indels), while base editing and prime editing systems remain underdeveloped (Komor et al., 2016; Tan et al., 2022; Xiang et al., 2025). In contrast, Cas9 and Cas12 have successfully integrated deaminases or reverse transcriptases, whereas similar tools for TnpB are still in their early stages of development. In terms of targeting range, TnpB’s DNA cleavage strictly depends on TAM sequences (e.g., 5′-TTGAT-3′or 5′-TTTAA-3′), significantly limiting the flexibility in selecting edit sites. Compared to CRISPR-Cas9 (PAM NGG) or Cas12f (PAM TTN), TnpB’s TAM appears less frequently across genomes, restricting its application in complex organisms like humans and plants. Recent protein engineering approaches (such as TnpBmax) have relaxed TAM restrictions by recognizing 5′-WNHNN-3′, yet their editing efficiency still lags behind Cas9 variants. This poses a major obstacle for research and applications requiring precise gene editing across extensive regions. The editing efficiency and off-target effects of TnpB remain key limitations hindering its development. Wild-type TnpB typically achieves only 0%–20% editing efficiency in mammalian cells, significantly lower than Cas9’s (>70%). Regarding off-target risks: Although TnpB’s RuvC domain demonstrates specificity, its potential for unintended cleavage has not been systematically evaluated. This risk is particularly concerning in eukaryotic cells, where non-standard TAM recognition may lead to unexpected gene cuts.

The expression of TnpB in host organisms also shows significant variations. Most TnpB systems (such as ISDra2 and SisTnpB1) were initially validated in prokaryotes (e.g., bacteria and archaea), but their suitability for eukaryotic cells (e.g., mammals and plants) still requires optimization. Additionally, efficient delivery of TnpB systems to target cells within the body remains a major challenge. Although TnpB’s compact structure theoretically offers advantages in *in vivo* delivery, practical implementation still faces difficulties in ensuring precise and efficient transport to specific cells while maintaining stable functionality. Current research on TnpB is predominantly confined to laboratory settings, with commercial applications limited to IDT’s Cas9 kit, which restricts its broader application.

In summary, the TnpB system faces significant limitations and challenges in development and application, including editing efficiency, targeting range, applicability, and *in vivo* delivery, which require further research and technical optimization. Future improvements to the TnpB system can be pursued through multiple approaches: First, modifying TnpB via directed evolution or computational protein design to enhance its TAM sequence compatibility, thereby expanding the number of gene editing sites. Second, addressing the low editing efficiency in eukaryotic cells by optimizing codon usage, enhancing nuclear localization signals, and applying AI prediction models to improve gRNA design strategies. Third, developing TnpB-based base editors by fusing TnpB with deaminases or reverse transcriptases to achieve more precise single-base editing. Finally, employing high-throughput methods like whole genome sequencing (WGS) or CIRCLE-seq to systematically evaluate TnpB’s off-target effects will ensure safety for clinical applications. Advancing these research directions will significantly enhance the TnpB system’s potential in gene editing.

## 5 Prospects

*Deinococcus radiodurans* exhibit extreme resistance to damage factors such as UV, drought and hydrogen peroxide, and their robust DNA repair mechanisms and efficient DNA uptake system make them attractive candidates for genetic engineering applications. The TnpB protein of *D. radiodurans* ISDra2 is believed to be the ancestor of the CRISPR nuclease Cas12f. The traditional CRISPR system has been limited in its application due to its large size. Although CRISPR-Cas12f is currently known as the smallest CRISPR system, it should be noted that while CRISPR-Cas12f is an effective and cost-efficient method for high-throughput pathogenic mutation screening, the sgRNA-mediated Cas12f targeted cleavage of ssDNA requires additional steps to generate ssDNA from dsDNA targets before detection. This particular characteristic of CRISPR-Cas12f could potentially increase the complexity of its application. Furthermore, the mechanism of action of TnpB protein and its related studies suggest that TnpB protein is expected to become a more convenient and practical gene editing tool in the future.

TnpB proteins are derived from mobile genetic elements such as transposons and are capable of inserting DNA sequences at specific genomic loci in a targeted manner. This integration process can be used to introduce exogenous DNA sequences, disrupt target genes or engineer specific genomic modifications. The natural diversity of TnpB homologs, including eukaryotic variants yet to be identified33 and their miniature size suitable for adeno-associated viral delivery, opens up new horizons for human therapeutic applications. Li Z. et al. (2024) achieved disease phenotype correction in tyrosinemia mouse models by modifying the ωRNA of ISDra2 TnpB, with no significant off-target effects detected through whole-genome analysis. This approach can be extended to treat other genetic disorders such as hypercholesterolemia and hemophilia, particularly suitable for scenarios requiring long-term expression editing tools (Li Z. et al., 2024). As with the Cas system, homology searches and phylogenetic trees allowed us to identify the TnpB system and understand its diversity. At the same time the predictability of TAMs is further enhanced, which promises to further expand the editing scope of TnpB. With CRISPR-Cas research as a foundation, TnpB is expected to break through the limitations of TAMs and become a smaller, simpler, and more practical gene editing tool in the future. Li Z. et al. (2024) achieved a significant boost in editing efficiency by progressively modifying the ωRNA of ISDra2 TnpB. This strategy can be further enhanced by integrating deep learning models to predict ωRNA sequences [as demonstrated by Marquart et al. (2024) model that increased activity by 4.4 times], enabling efficient editing of complex genomic sites (such as oncogenes with repetitive sequences). A research team from the Institute of Zoology, Chinese Academy of Sciences and the School of Life Sciences at Southwest University has identified TnpB proteins including ISAam1 (369 amino acids), ISYmu1 (382 amino acids), and IsDge10 through genome mining of prokaryotic genomes. These proteins demonstrated comparable editing efficiency to SaCas9 in both human cells and rice, surpassing some Cas12f proteins and their variants (Xiang et al., 2024). Further screening of high-activity TnpB nuclease enzymes and optimization of their editing performance will facilitate the development of compact, efficient, and precise gene-editing tools to meet the demands of gene therapy and plant genetic improvement.
